# Supplementary material for: Resolving the full spectrum of human genome variation using Linked-Reads
Source: Genome Res. 2019 Apr;29(4):635–45. doi: 10.1101/gr.234443.118 (PMC6442396; doi:10.1101/gr.234443.118)
Supplement: Supplemental Material [file supp_gr.234443.118_Supplemental_longranger-2.2.zip › longranger-2.2.tar.bz2!/lr2.2-public/lib/stan/cmdstan/stan/doc/api/html/check__less__or__equal_8hpp_source.html]

Stan: src/stan/math/error\_handling/check\_less\_or\_equal.hpp Source File


|  |  |
| --- | --- |
|  | Stan  2.5.0  probability, sampling & optimization |


- Main Page
- Related Pages
- Namespaces
- Classes
- Files

- File List
- File Members

All Classes Namespaces Files Functions Variables Typedefs Enumerations Enumerator Friends Macros Pages

- src
- stan
- math
- error\_handling

check\_less\_or\_equal.hpp

Go to the documentation of this file.

1 #ifndef STAN\_\_MATH\_\_ERROR\_HANDLING\_CHECK\_LESS\_OR\_EQUAL\_HPP

2 #define STAN\_\_MATH\_\_ERROR\_HANDLING\_CHECK\_LESS\_OR\_EQUAL\_HPP

3

4 #include <stan/math/error\_handling/dom\_err.hpp>

5 #include <stan/math/error\_handling/dom\_err\_vec.hpp>

6

7 namespace stan {

8  namespace math {

9

10  namespace {

11  template <typename T\_y, typename T\_high, typename T\_result, bool is\_vec>

12  struct less\_or\_equal {

13  static bool check(const char\* function,

14  const T\_y& y,

15  const T\_high& high,

16  const char\* name,

17  T\_result\* result) {

18  using stan::length;

19  VectorView<const T\_high> high\_vec(high);

20  for (size\_t n = 0; n < length(high); n++) {

21  if (!(y <= high\_vec[n]))

22  return dom\_err(function,y,name,

23  " is %1%, but must be less than or equal to ",

24  high\_vec[n],result);

25  }

26  return true;

27  }

28  };

29

30  template <typename T\_y, typename T\_high, typename T\_result>

31  struct less\_or\_equal<T\_y, T\_high, T\_result, true> {

32  static bool check(const char\* function,

33  const T\_y& y,

34  const T\_high& high,

35  const char\* name,

36  T\_result\* result) {

37  using stan::length;

38  VectorView<const T\_high> high\_vec(high);

39  for (size\_t n = 0; n < length(y); n++) {

40  if (!(stan::get(y,n) <= high\_vec[n]))

41  return dom\_err\_vec(n,function,y,name,

42  " is %1%, but must be less than or equal to ",

43  high\_vec[n],result);

44  }

45  return true;

46  }

47  };

48  }

49

50  // throws if any element of y or high is nan

51  template <typename T\_y, typename T\_high, typename T\_result>

52  inline bool check\_less\_or\_equal(const char\* function,

53  const T\_y& y,

54  const T\_high& high,

55  const char\* name,

56  T\_result\* result) {

57  return less\_or\_equal<T\_y,T\_high,T\_result,is\_vector\_like<T\_y>::value>

58  ::check(function,y,high,name,result);

59  }

60

61  }

62 }

63 #endif

dom\_err.hpp

stan::math::dom\_err

bool dom\_err(const char \*function, const T &y, const char \*name, const char \*error\_msg, const T\_msg error\_msg2, T\_result \*result)

**Definition:** dom\_err.hpp:36

stan::length

size\_t length(const T &)

**Definition:** traits.hpp:159

stan::math::dom\_err\_vec

bool dom\_err\_vec(const size\_t i, const char \*function, const T &y, const char \*name, const char \*error\_msg, const T\_msg error\_msg2, T\_result \*result)

**Definition:** dom\_err\_vec.hpp:33

stan::math::check\_less\_or\_equal

bool check\_less\_or\_equal(const char \*function, const T\_y &y, const T\_high &high, const char \*name, T\_result \*result)

**Definition:** check\_less\_or\_equal.hpp:52

dom\_err\_vec.hpp

stan::get

T get(const T &x, size\_t n)

**Definition:** traits.hpp:143


---

[ Stan Home Page ]

*© 2011–2014,
Stan Development Team.*
